# Chronic suppurative otitis media: Socio-economic implications in a tertiary hospital in Northern Nigeria

**DOI:** 10.4314/pamj.v4i1.53613

**Published:** 2010-01-26

**Authors:** Adeyi Adoga, Tonga Nimkur, Olugbenga Silas

**Affiliations:** 1 Otorhinolaryngology Unit, Department of Surgery, Jos University Teaching Hospital, PMB 2076, Jos, Plateau State, Nigeria.; 2 Department of Pathology, Jos University Teaching Hospital, PMB 2076, Jos, Plateau State, Nigeria.

**Keywords:** Socioeconomic Implications, Chronic Suppurative Otitis Media, Nigeria

## Abstract

**Background:**

In developing countries, ear infections and deafness are usually neglected conditions due to insufficient funds, work force, facilities, and knowledge. This paper highlights the socio-economic burden of chronic suppurative otitis media on a northern Nigerian population with suggestions on ways to reduce this burden.

**Method:**

Seventy-four patients presenting to the Otorhinolaryngology unit, Department of surgery, Jos University Teaching Hospital, Plateau state, Nigeria with chronic suppurative otitis media from June 2007 to May 2008 were evaluated for age, gender, occupation, otomicroscopy and audiologic findings, microscopy, culture and sensitivity results, cost of consultation, investigations and treatment, type of complications and the social impact on individuals.

**Results:**

Patients were aged 2 to 37 years (Mean age=9.23 years, SD=7.92). Forty-one (55.4%) patients were aged between 1 and 5 years. There were 8 (10.8%) students, 12 (16.2%) unskilled workers, 11 (14.9%) unemployed individuals and 2 (2.7%) professionals. Deafness was the commonest sequel. Minimum monthly wage was 7,500 ($47.5US). Initial cost of treatment per patient per year was 8,100 Naira ($51.3US) increasing to 73,100 Naira ($462.7US) if surgery and hearing aid was required. Eleven (15%) patients required surgery, only 2 (2.7%) patients could afford it. Four patients lost their jobs. Fourteen (18.9%) patients were lost to follow up.

**Conclusion:**

The estimated cost of treatment for chronic suppurative otitis media is higher than the monthly minimum wage for individuals in our environment where the cost of health care is the sole responsibility of the patient.

## Introduction

Chronic suppurative otitis media (CSOM), a phenomenon virtually non-existent in the developed world, still constitutes a major public health problem in children and adults in Africa, Asia and Latin America [1]. It is an infection characterized by recurrent middle ear discharge through a persistent tympanic membrane perforation, which can be managed at the primary health care level thereby preventing the development of deafness and even fatal complications [2]. Commonly a disease of the developing world with malnutrition, over-crowding, substandard hygiene, frequent upper respiratory tract infections and under-resourced health care (all linked to low socio-economic status) listed as risk factors [1, 3]. The poorer rural communities have the highest prevalence [4].

It is the commonest childhood infectious disease worldwide [5] starting early in life but in our environment, presentation may be in adult life [6]. Individuals in our environment tend to live with the disease, tolerate its discomfort with resultant fatal consequences not only because of insufficient work force and health care facilities but also due to inaccessibility to health care [1]. When established, it is very difficult to treat. Medical management needs to be continued for many weeks and even when the perforation is dry; patients are at risk of further episodes of discharge until the tympanic membrane has healed [7].

The infection can spread from the middle ear to involve the mastoid, facial nerve, labyrinth, lateral sinus, meninges and brain leading to mastoid abscesses, facial nerve paralysis, deafness, lateral sinus thrombosis, meningitis and intracranial abscesses [8]. Of all these complications, hearing loss associated with the chronic discharging ear is nearly always significant, reported in 50% of cases and tending to be more severe than those associated with other types of otitis media [7]. Which means affected individuals will need additional audiological and educational support.

The World Health Organization (WHO) global estimate for disabling hearing impairment (degree of severity more than 40 dB) has more than doubled from 120 million people in 1995 to 278 million in 2005 [9, 10]. A total of 364 million people have mild hearing impairment, while 624 million are estimated to have some level of hearing impairment and 80% of these live in low and middle-income countries [10].

The aims of managing the chronic discharging ear are early detection and timely, appropriate intervention to eradicate the disease permanently or to reduce its effects (i.e., ear discharge, hearing loss and other complications) if eradication is not possible [1]. This can be solved by regular aural toileting, antibiotic treatment, middle ear reconstruction and the use of hearing aids for rehabilitation. In sub-Saharan Africa, this will seem an arduous task due to poverty and the task of prioritizing health care needs in the face of limited and diminishing resources [11].

The aim of this paper is to highlight the socio-economic burden of this disease on a northern Nigerian population with suggestions on ways to reduce this burden.

## Methods

After obtaining approval from the Ethical Committee of the Jos University Teaching Hospital, 74 patients who presented to the otorhinolaryngology unit of the Jos University Teaching Hospital with chronic suppurative otitis media from June 2007 to May 2008 were evaluated for age, gender, occupation and duration of illness. Other parameters evaluated include findings at otomicroscopy, audiologic findings and results from microscopy, culture and sensitivity, the economic cost of consultation, investigations, treatment, the type of complications seen and the social impact on individuals. There is a listed amount for each type of health care service rendered in our teaching hospital. These costs for consultation, investigations and treatment were used in our study to calculate the mean cost per patient per year. Patients were followed up throughout the study period and beyond for treatment outcome and possibility of development of complications.

## Results

Seventy-four new patients with chronic suppurative otitis media were seen aged between 2 and 37 years (Mean age=9.23 years, SD=7.92), accounting for 31.1% of all otologic presentations and 15.2% of all new cases in the study period. There were 47 (63.5%) males and 27 (36.5%) females with a Male: Female ratio of 1.6:1.

In our study, 55.4% of the patients were aged between 1 and 5 years (Table 1). There were 8 (10.8%) students, 12 (16.2%) unskilled workers, 11 (14.9%) unemployed individuals and 2 (2.7%) professionals. Twelve (16.2%) patients’ occupations were not listed. The duration of symptoms ranged from 8 months to 17 years.

**Table 1:** Age distribution of patients with CSOM

Age(years)	Frequency	Percentage
1–5	41	55.4
6–10	10	13.5
11–15	8	10.8
16–20	5	6.7
21–25	7	9.5
26–30	0	0
31–35	2	2.7
36–40	1	1.4
**Total**	**74**	**100**

Otomicroscopy revealed unilateral disease in 46 (62.2%) and bilateral disease in 28 (37.8%) patients. Seventy-two (97.3%) patients had tubotympanic disease while 2 (2.7%) patients had attico-antral disease. Fifty-eight (78.4%) patients had otorrhea at presentation while 16 (21.6%) did not. All the patients with otorrhea had ear swabs taken for microscopy, culture and sensitivity (MCS). Treatment was by regular aural toileting and alternate daily ear wick dressings with quinolone antibiotics (containing steroids) for a minimum of 2 weeks and a maximum of 4 weeks and the use of decongestants. The average number of clinic visits is 10 per year.

Thirty-five (47.3%) patients had persistent dry ear following ear wick dressings and systemic decongestants during follow up in the study period. Follow up of patients continues. The main pathogens on MCS were Pseudomonas aeruginosa, Staphylococcus aureus, Klebsiella and Escherichia coli, and their sensitivity rates for ciprofloxacin were 89.0%, 88.2%, 84.0% and 85.0% respectively. Audiometry was done and hearing loss was recorded in all the patients- mild hearing loss in 49 (66.2%), moderate in 22 (29.7%) and severe in 3 (4.1%) patients.

The complications noted (Table 2) were mastoid abscess in 5 (6.8%), subperiosteal abscess in 1 (1.4%), meningitis in 1 (1.4%) and facial nerve paralysis in 1 (1.4%) patients. Eleven (15%) patients required surgery but only 2 (2.7%) patients successfully had middle ear reconstruction (Table 3).

**Table 2:** Sequel of Chronic suppurative otitis media

Complication	Frequency	Percentage
Hearing loss	74	100
Mastoid abscess	5	6.8
Subperiosteal abscesses	1	1.4
Meningitis	1	1.4
Facial nerve palsy	1	1.4

**Table 3:** Cost of hospital visits, diagnosis and treatment per Chronic suppurative otitis media patient per year

Service rendered	Cost of service (Naira)
Registration/initial consultation	150
Follow-up visits (average of 10 visits/year)	750
Ear swab MCS	500
Pure tone audiometry (PTA)	1,500
Mastoid imaging	2,700
Medical treatment (ear dressings & decongestants)	2,500
Surgery/hospitalization	35,000
Hearing aid	30,000
**Total**	**73,100 ($462.7US)**

The recorded financial cost per patient for initial treatment was 8,100 Naira ($51.3US). For patients requiring surgery/hospitalization and hearing aids this increases to 73,100 Naira ($462.7US).

Five (6.8%) patients could afford and benefitted from hearing aids. Four (28.6%) patients lost their jobs because of the impact of the disease on their ability to work. Fourteen (18.9%) patients were lost to follow up.

## Discussion

Chronic suppurative otitis media (CSOM) is an infection commonly associated with poor socio-economic status or poverty-related conditions such as malnutrition, over-crowding, substandard hygiene, frequent upper respiratory tract infections and under-resourced health care [1].

Like in other studies, our study shows that children are the most affected by this disease, especially the under-fives [5, 12], accounting for 31.1% of all otologic presentations and 15.2% of all new cases in our study. These are astonishing figures considering the fact that this disease is preventable. Experts declare that when the prevalence of CSOM is 3% or greater, it must be targeted as a high priority disease [13]. In many developing countries, Nigeria inclusive, prioritizing health care needs is a difficult task in the face of limited and diminishing resources. Therefore achieving the Millennium Development Goals (MDGs) for these countries is almost impossible without the assistance of donor organizations like the World Health Organization (WHO), the World Bank, United Nations Children’s Fund (UNICEF) and the Rotary International to mention a few. The health focus of these countries then becomes those prescribed by these donor agencies with neglect of important disease conditions like CSOM [11].

Tubotympanic disease was more prevalent than attico-antral disease in our study. Attico-antral is rare among Nigerians and the reason given for this is a good Eustachian tube function [6, 14]. Reducing the burden of hearing loss is another worthwhile goal even as attempts are made to prevent CSOM. All the patients in this study had various types of hearing impairment, which is a significant cause of disability. This disability was the reason for the loss of jobs recorded in four of our patients, a cost which is unquantifiable for each individual and their various families.

While middle ear reconstruction may be advised for any patient with CSOM, hearing amplification through the provision of hearing aids is also a worthwhile alternative for those who desire hearing rehabilitation. The high costs of hearing aids (the least cost in our environment being about 35,000 Naira i.e. $221.5US) and the stream of costs to which patients using hearing aids are liable, may still make surgical reconstruction a more cost effective alternative despite variable success rates and anesthetic risks. Even this is made difficult by the private funding of health services that accounts for about 75% of total health expenditure in many developing countries.

The recorded financial cost per patient for the initial treatment of this disease in our study was 8,100 Naira i.e. $51.3US. This amount further increased to 73,100 Naira ($462.7US) for patients requiring middle ear exploration and hearing aids, which is expensive considering CSOM is a disease of low socio-economic class and in our country where the minimum monthly wage is 7,500 Naira ($47.5US) this is a significant burden. This factor tends to select individuals based on their ability to pay for services and is a possible deterrent to the development and provision of services for such conditions as hearing health care [15]. The finding in this study, whereby only two out of nine patients were able to afford middle ear reconstruction and these patients were of the high-income group, further supports this statement.

Proper and effective enforcement of the National Health Insurance Scheme (NHIS) in our environment can go a long way in helping low and medium income earners to afford proper health care. Fourteen patients were lost to follow up in our study. This is a common feature in our environment and is due to several factors such as patients not seeing the need for further review in the hospital as they achieve dry ears, lack of financial capability for further hospital visits, or the patients seeking help elsewhere with the herbalist.

The encouraging news is that CSOM can be controlled at the primary health care level with the prevention of fatal consequences. This can be achieved if patients have rapid access to primary health care where adequately trained, skilled community health workers, nurses and doctors are available [2]. The WHO Primary Ear and Hearing Care Training Resource is helping in this regard [16] but these efforts should be intensified in Nigeria to reduce the burden of this disease.

With the evolution of Public-Private partnership as an alternative funding strategy for low and middle income countries due to the inability of their governments to provide diverse health needs from limited or improperly executed budgets, some of these health care needs have been met. An example is the support of individuals with hearing loss by charitable organizations such as the Rotary International, Lions Club via the provision of hearing aids amongst other things [11].

The recent release of funds by the International Finance Corporation, the private sector arm of the World Bank to the private sector in Africa to improve health care services, is a welcome development [17]. The global partnership of the Worldwide Hearing (WWH) Care for Developing Countries and the WHO are also working towards the provision of affordable hearing aids and services to governments in developing countries [10].

Chronic suppurative otitis media like any chronic disease, can limit an individual’s employability and quality of life. They are particularly disadvantaged because of scarcity of work, poor living conditions and limited health care [1]. As long as health care delivery fails to target high-risk groups in developing countries, infections like CSOM will persist. Therefore, improving equal access to good health care and ensuring health care programs respond appropriately to individual health needs is a critical factor to ultimately ridding the world of this disease.

## Conclusion

The estimated cost of initial treatment for chronic suppurative otitis media of 8,100 Naira ($462.7US) which increases to 73,100 Naira ($51.3US) if surgery and hearing aid is required is rather high for individuals in a country where the monthly minimum wage is 7,500 Naira ($47.5US) and the cost of health care is the sole responsibility of the patient.

## Acknowledgements

Our appreciation goes out to Dr. (Mrs.) M. E. Banwat for her assistance in data analysis.

## Competing interests

The authors declare that they have no competing interests. This paper was presented at the 49th Annual Scientific Conference of the West African College of Surgeons in Conakry, Guinea in February 2009.

## Authors’ contributions

**AA**: Conceived of this work, collected and analyzed data, performed literature search and prepared the manuscript.

**TN**: Collected and analyzed data and reviewed the manuscript.

**OS**: Performed literature search and reviewed the manuscript.

## References

[1] Acuin JM (2007). Chronic suppurative otitis media: a disease waiting for solutions. Comm Ear Hearing H.

[2] van Hasset P (2007). Chronic suppurative otitis media. Comm Ear Hearing H.

[3] Lasisi AO, Sulaiman OA, Afolabi OA (2007). Socio-economic status and hearing loss in chronic suppurative otitis media in Nigeria. Ann Trop Paediatr.

[4] Ologe FE, Nwawolo CC (2003). Chronic suppurative otitis media in school pupils in Nigeria. East Afr Med J.

[5] Verhoeff M, van der Veen EL, Rovers MM, Sanders EA, Schilder AG (2006). Chronic suppurative otitis media: a review. Int J Pediatr Otorhinolaryngol.

[6] Okafor BC (1984). The chronic discharging ear in Nigeria. J Laryngol Otol.

[7] Morris PS, Leach AJ (2007). Prevention and management of chronic suppurative otitis media in aboriginal children: a practical approach. Comm Ear Hearing H.

[8] Berman S (1995). Otitis media in developing countries. Pediatrics.

[9] Olusanya BO, Newton VE (2007). Global burden of childhood hearing impairment and disease control priorities for developing countries. Lancet.

[10] Smith AW

[11] Olusanya BO, Newton VE (2007). Promoting a global health agenda for permanent childhood hearing impairment. Comm Ear Hearing H.

[12] Ologe FE, Nwawolo CC (2002). Prevalence of chronic suppurative otitis media (CSOM) among school children in a rural community in Nigeria. Niger Postgrad Med J.

[13] World Health Organization

[14] Okafor BC (1978). The discharging ear in Nigeria. Nig Med J.

[15] White KR (2006). The role of the JCIH in the global expansion of newborn hearing screening. J Am Acad of Audiol.

[16] (2006). Primary Ear and Hearing Care Training Resource.Advanced level.WHO.

[17] (2007). The Economist.Healthcare in Africa: of markets and medicines. Economist.

